# Longitudinal monitoring of EGFR mutations in plasma predicts outcomes of NSCLC patients treated with EGFR TKIs: Korean Lung Cancer Consortium (KLCC-12-02)

**DOI:** 10.18632/oncotarget.6874

**Published:** 2016-01-09

**Authors:** Ji Yun Lee, Xu Qing, Wei Xiumin, Bai Yali, Sangah Chi, So Hyeon Bak, Ho Yun Lee, Jong-Mu Sun, Se-Hoon Lee, Jin Seok Ahn, Eun Kyung Cho, Dong-Wan Kim, Hye Ryun Kim, Young Joo Min, Sin-Ho Jung, Keunchil Park, Mao Mao, Myung-Ju Ahn

**Affiliations:** ^1^ Division of Hematology-Oncology, Department of Medicine, Samsung Medical Center, Sungkyunkwan University School of Medicine, Seoul, Korea; ^2^ Translational Bioscience and Diagnostics, WuXi AppTec, Waigaoqiao Free Trade Zone, Shanghai, China; ^3^ Department of Biostatistics and Bioinformatics, Samsung Medical Center, Sungkyunkwan University School of Medicine, Seoul, Korea; ^4^ Department of Radiology, Kangwon National University Hospital, Chuncheon, Korea; ^5^ Department of Radiology, Center for Imaging Science, Samsung Medical Center, Sungkyunkwan University School of Medicine, Seoul, Korea; ^6^ Division of Hematology-Oncology, Department of Medicine, Gachon Medical School, Gil Medical Center, Inchon, Korea; ^7^ Division of Hematology-Oncology, Department of Internal Medicine, Seoul National University Hospital, Seoul, Korea; ^8^ Division of Oncology, Department of Medicine, Yonsei University College of Medicine, Seoul, Korea; ^9^ Department of Oncology, Asan Medical Center, University of Ulsan college of Medicine, Seoul, Korea

**Keywords:** NSCLC, EGFR, TKI treatment, droplet digital PCR, liquid biopsy

## Abstract

We hypothesized that plasma-based EGFR mutation analysis for NSCLC may be feasible for monitoring treatment response to EGFR TKIs and also predict drug resistance. Clinically relevant mutations including exon 19 deletion (ex19del), L858R and T790M were analyzed using droplet digital PCR (ddPCR) in longitudinally collected plasma samples (*n* = 367) from 81 NSCLC patients treated with EGFR TKI. Of a total 58 baseline cell-free DNA (cfDNA) samples available for ddPCR analysis, 43 (74.1%) had the same mutation in the matched tumors (clinical sensitivity: 70.8% [17/24] for L858R and 76.5% [26/34] for ex19del). The concordance rates of plasma with tissue-based results of EGFR mutations were 87.9% for L858R and 86.2% for ex19del. All 40 patients who were detected EGFR mutations at baseline showed a dramatic decrease of mutant copies (>50%) in plasma during the first two months after treatment. Median progression-free survival (PFS) was 10.1 months for patients with undetectable EGFR *v* 6.3 months for detectable EGFR mutations in blood after two-month treatment (HR 3.88, 95% CI 1.48-10.19, *P* = 0.006). We observed emerging resistance with early detection of T790M as a secondary mutation in 14 (28.6%) of 49 patients. Plasma-based EGFR mutation analysis using ddPCR can monitor treatment response to EGFR TKIs and can lead to early detection of EGFR TKIs resistance. Further studies confirming clinical implications of EGFR mutation in plasma are warranted to guide optimal therapeutic strategies upon knowledge of treatment response and resistance.

## INTRODUCTION

Non-small cell lung cancer (NSCLC) has a dismal prognosis with one of the highest mortality rates among cancer types [1, 2]. Patients with NSCLC harboring epidermal growth factor receptor (EGFR) mutation respond to treatment of EGFR tyrosine kinase inhibitors (TKIs) [3, 4]. Large randomized phase III trials comparing EGFR TKIs such as gefitinib, erlotinib or afatinib with cytotoxic chemotherapy consistently demonstrated higher response rates and prolonged progression-free survival (PFS) in EGFR mutant NSCLC patients, leading to the standard treatment of EGFR TKI as first line therapy [5-8]. However, most patients with NSCLC treated with EGFR TKIs eventually develop acquired resistance [9, 10]. Although multiple mechanisms are involved, EGFR T790M mutation accounts for more than 50% of the acquired TKI resistance [11, 12]. Detection of resistance requires acquisition and analysis of tumor tissue at the time of resistance to understand the underlying mechanism. However, the invasive nature of repeated biopsies makes it difficult to obtain from patients particularly those with poor performance. Other limitations include tumor heterogeneity [13], inaccessibility of re-biopsy due to tumor location or tumor containing blood vessel or air bronchogram, high incidence of tumor necrosis, and a single snapshot in time unable to track dynamic changes in the mutation.

The circulating tumor DNA in plasma with its non-invasive technology has been used as a surrogate for tumor tissues in detecting genetic alterations, thus providing molecular evolution of the tumors [14-17]. Bai et al. showed the difference in response rates (59.5% and 23.1%, *P* = 0.002) for gefitinib between patients with and without activating EGFR mutation in plasma [18]. Recently, droplet digital polymerase chain reaction (ddPCR), a highly sensitive technology has been developed for detecting low frequency cancer-associated mutations [19]. We hypothesized that plasma-based EGFR mutation analysis using ddPCR may be feasible in monitoring response and resistance to EGFR TKIs. Therefore, we conducted a multi-center prospective study to assess dynamic changes in EGFR mutation profile using a ddPCR method in longitudinally collected plasma samples from NSCLC patients harboring activating EGFR mutations treated with EGFR TKIs.

## RESULTS

### Patient characteristics

From January 2012 to October 2014, 81 EGFR mutant NSCLC patients who were treated with gefitinib or erlotinib and eventually developed acquired resistance were enrolled. The median age was 58.2 years (range, 32.1-81.1 years), 61.7% were female, and 63.0% were never smokers. Over four-fifths of patients (84.0%) had stage IV disease, 59.3% had ex19del and 59.3% were treated with EGFR TKIs as first-line therapy (Table 1).

**Table 1:** Baseline characteristics (n = 81)

	No. of patients	%
Age, years		
Median	58.2	
Range	32.1-81.1	
Sex		
Male	31	38.3
Female	50	61.7
Smoking history		
Never smoker	51	63.0
Former smoker	18	22.2
Current smoker	12	14.8
Tumor type		
Adenocarcinoma	80	98.8
Adenosquamous	1	1.2
Tumor stage		
IV	68	84.0
Postoperative relapse	13	16.0
Type of EGFR mutation		
Exon 19 deletion	48	59.3
L858R	33	40.7
Line of EGFR TKIs		
First line	48	59.3
Second line	33	40.7
EGFR TKIs		
Gefitinib	58	71.6
Erlotinib	23	28.4

Abbreviations: EGFR, epidermal growth factor receptor; TKIs, kinase inhibitors

### Clinical outcomes

Of total 81 patients, 59 (72.8%) patients achieved an objective response. With a median follow-up of 18.8 months, the median PFS and overall survival (OS) were 8.1 months (95% CI, 6.2-10.0) and 23.5 months (95% CI, 19.4-27.6), respectively.

### The technical sensitivity and specificity of L858R and ex19del assays in the cfDNA

**Figure 1:**
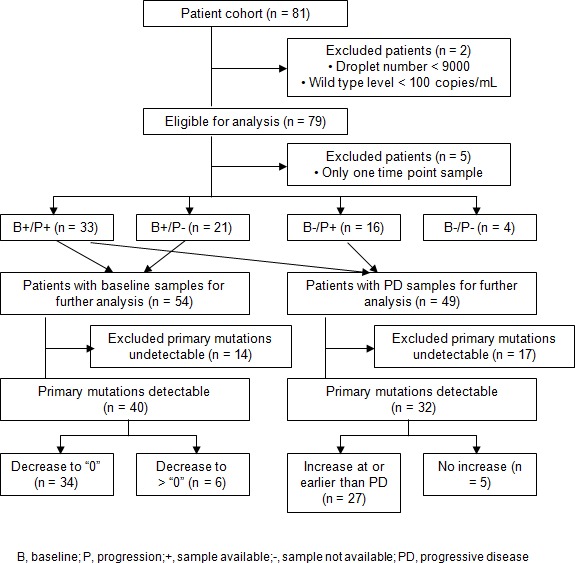
Patients exclusion in the analysis based on the availability of the baseline and PD samples, as well as primary mutations (L858R, ex19del) detection status (“detectable” vs. “undetectable”)

The sensitivity and specificity of the ddPCR assays in measuring EGFR mutations in cfDNA were determined from 367 plasma samples collected from baseline to every 8 weeks after EGFR TKI treatment from 81 patients. We applied two QC criteria for the results of these plasma samples: 1) the droplet number must be greater than 9000; 2) the wild type (WT) levels to be greater than 100 copies/mL. After QC, we excluded two patients and 6 time points, resulting in 361 samples from 79 patients (Figure 1). Among the qualified samples, the lowest level of WT cfDNA was 1040 copies/mL, and the median was 4568 copies/mL (data not shown), suggesting that good quality of cfDNA is obtained with the current sample preparation procedures.

**Figure 2:**
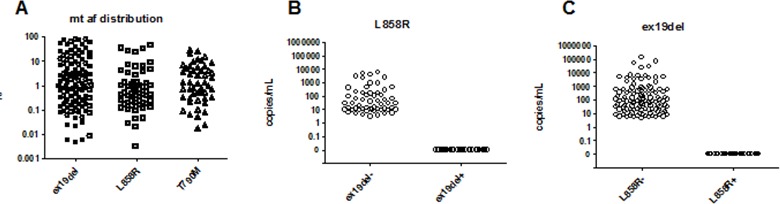
Technical sensitivity and specificity of cell-free plasma DNA using ddPCR assay (n = 79) **A**. distribution of frequency of alleles according to the EGFR mtuations (ex19del, L858R, and T790), **B**. detection of L858R mutant alleles in ex19del- negative and ex19del- positive population, and **C**. detection of ex19del mutant alleles in L858R-negative and L858R-positive population.

Since ddPCR assays are very sensitive in quantifying rare mutation events, we were able to detect very low MT allele frequency (AF) in the cfDNA for both L858R and ex19del mutations. The lowest MT AF detected among 57 L858R+ samples was 0.003%, while among 113 ex19del+ samples was 0.005% (Figure 2A). To test the specificity, we analyzed the results based on the mutual exclusivity of the two mutations in the same sample. For the L858R assay, we observed no L858L mutant copies in the 110 ex19del+ plasma samples (Figure 2B); for ex19del assay, no ex19del mutant copies were detected in the 57 L858R+ samples (Figure 2C), which demonstrated 100% of specificity.

### Clinical sensitivity of plasma-based EGFR mutation assays and their concordances with tissue mutation status

The clinical sensitivity of the EGFR mutation detection in plasma was assessed among 58 patients whose baseline samples were available. L858R or ex19del mutations were detected in 43 patients (Table 2) yielding sensitivity of 74.1% (43/58); 70.8% (17/24) for L858R and 76.5% (26/34) for ex19del. We assessed the specificity based on the mutual exclusivity of L858R mutation and ex19del mutation in EGFR mutant tumors. No L858R mutation was detected in the ex19del patient plasmas, and vice versa. Therefore, the specificity of cfDNA L858R assay and ex19del assay was 100% (34/34) and 100% (24/24) respectively. The concordance rate between cfDNA and tissue was 87.9% for L858R and 86.2% for ex19del before treatment.

**Table 2:** Clinical sensitivity and concordance between tissue and cfDNA measurement results

	Prior Treatment (*n* = 58)						Disease Progression (*n* = 49)					
	cfDNA						cfDNA					
Tissue	L858R+	L858R−	Total	Ex19del+	Ex19del−	Total	L858R+	L858R−	Total	Ex19del+	Ex19del−	Total
Positive	17	7	24	26	8	34	11	10	21	21	7	28
Negative	0	34	34	0	24	24	0	28	28	0	21	21
Total	17	41	58	26	32	58	11	38	49	21	28	49
Sensitivity	70.8			76.5			73.7			75.0		
Specificity	100			100			100			100		
Concordance	87.9			86.2			79.6			85.7		

Abbreviations: cfDNA, cell-free DNA; Ex19del, exon 19 deletion

We then examined the EGFR mutation detection rate in plasma from patients who experienced progressive disease (PD). Of 79 patients, 49 patients had plasma samples available at the time of progression. Among 49 patients, L858R and ex19del mutations were detected in 32 patients, resulting in 65.4% (32/49) clinical sensitivity in plasma at disease progression (Figure 1, Table 2).

We subsequently assessed the detection rate of resistance mutation T790M among patients who developed PD. As described previously [20], the technical background of T790M assay in fresh and FFPE gDNA is 0.03% and 0.05%, respectively. The quadruplicate repeats of the test were implemented in determining the true positives of plasma T790M measurements. Among the four data points for each sample, only those with at least three non-zero readings were considered as positives. With these criteria, 50 out of 361 samples were positive in the T790M detection. The lowest allele frequency detected was 0.017% and the median was 1.24% (Figure 2A). Among 49 patients with available samples at the time of progression, 14 patients (28.6%) had detectable T790M in their plasma.

### Longitudinal monitoring for primary and resistance EGFR mutations in patients treated with EGFR TKIs

To investigate whether cfDNA mutation level can be used to monitor the treatment response and resistance longitudinally, we analyzed plasma samples at specified times over the course of treatment cycle. Among 79 patients, five patients who had only one time point and four who had neither baseline nor PD data points were excluded for analysis (Figure 1). In addition, 24 samples collected beyond disease progression were excluded. Among 70 patients with more than 1 available time point, 33 patients had both baseline and PD points, 21 patients had only baseline, and 16 had only PD (Figure 1, Table 3).

**Table 3:** Dynamic monitoring of patient response to therapy by cfDNA

Patient	Total (*n*=70*)	cfDNA mutation at baseline (n=40) L858R/ex19del	Dynamic monitoring of cfDNA mutation					
			L858R/ex19del				T790M	
			Decrease not zero	Decrease to zero	Increase at progression	Increase earlier than progression	Increase at progression	Increase earlier than progression
B+P+	33	24	3	21	10	10	4	5
B+P-	21	16	3	13				
B-P+	16				5	2	2	3

Abbreviations: cfDNA, cell-free DNA; Ex19del, exon 19 deletion

Available samples: B+, available at baseline; P+, available at progression

* Among the 79 patients, 5 patients who had only one time point and 4 patients who had neither baseline nor PD data point data excluded for time course analysis.

**Figure 3:**
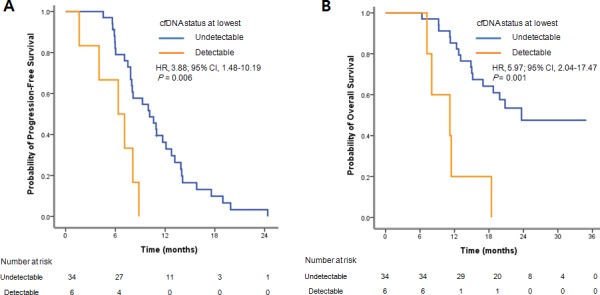
Survival curves for the 40 patients treated with EGFR TKIs **A**. Progression-free survival (PFS) and **B**. overall survival (OS) by cfDNA status at lowest level during the treatment.

The level of L858R copies at baseline were shown to be significantly (*P* = 0.0068) associated with shorter PFS. But we did not see the correlation in ex19del (Supplementary Figure 1). Among the 54 patients whose baseline samples were available for analysis, L858R or ex19del mutations were detected in 40 patients’ plasmas at the baseline. All 40 patients showed decreased mutant DNAs in circulation after EGFR TKI treatment. Of note, 34 patients (85.0%) had plasma EGFR mutations decreased to the undetectable level (0 copy) during the treatment cycle compared to the baseline (Table 3, Supplementary Figure 2). Intriguingly, there was a significant difference in PFS between patients with undetectable EGFR mutation and detectable EGFR mutation (10.1 months *vs* 6.3 months; HR 3.88, 95% CI 1.48-10.19, *P* = 0.006; Figure 3A). Similarly, the median OS of patients with undetectable EGFR and detectable EGFR were 23.7 and 11.2 months, respectively (HR 5.97, 95% CI 2.04-17.47, *P* = 0.001; Figure 3B). Of 49 patients with PD plasma samples, 15 patients (30.6%) showed increased mutation level at the PD time point, and 12 patients (24.5%) showed increased mutation level about 2 to 12 months earlier than the PD time point, as judged by the primary mutation L858R or ex19del (Figure 1, Table 3).

Among 49 patients with available samples at the time of progression, the T790M mutation was detected in 8 patients (16.3%) as early as 2 to 12 months prior to radiological progression: the remaining 6 patients (12.2%) developed T790M mutation at the time of PD (Supplementary Figure 3). It is noteworthy that one patient had detectable prior treatment T790M mutation. Although plasma ex19del and T790M levels were decreased with clinical response after gefitinib treatment in patients with de novo T790M mutation, brain and bone metastases occurred after 6 months of gefitinib treatment.

**Figure 4:**
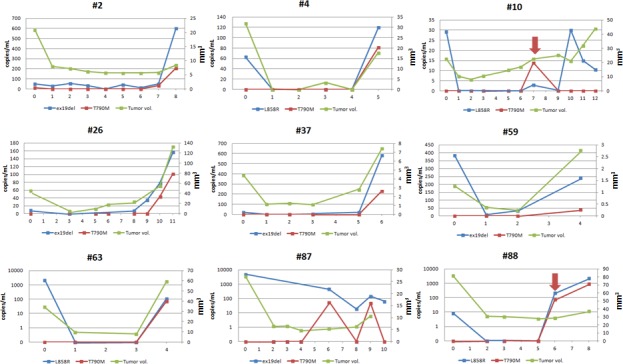
Longitudinal L858R, ex19del and T790M levels in plasma along with tumor volume measured by CT scan Arrow (red) shows increased copy number of both plasma L858R and T790M before RECIST-defined progression in two patients (#10 and #88). (Unit of x-axis: time point of acquisition of plasma (every 8 weeks). 0 = baseline).

The tumor volume was assessed using Chest CT scans by radiologists (HY Lee, and SH Bak) for each patient every eight weeks. Figure 4 shows the levels of circulating two EGFR primary mutations and the corresponding tumor volumes over the course of treatment to disease progression. In patients whose plasma T790M mutation was detected at the time of progression, T790M was generally at slightly lower levels than the primary mutations. In one patient (#2) with a response to gefitinib, an increase in plasma ex19del and T790M levels was seen during the development of bone metastasis (Figure 4). Two patients (#10 and #88) showed an increase in copy number of both plasma L858R and T790M before RECIST-defined progression, 11 months and 2 months, respectively (Figure 4).

## DISCUSSION

The development of acquired resistance to anticancer therapy derives from clonal evolution and selection [21, 22]. Thus, serial biopsies for the tumor are required to identify the underlying drug resistance mechanism and potential therapeutics to overcome or prevent it. Moreover, monitoring treatment response accompanied by mutations in the tumor genome may be needed to evaluate the potential benefits of targeted cancer therapies and subsequently determine and tailor optimal treatment strategies for patients. However, detection of EGFR mutations in NSCLC patients using sampling tumor tissues has significant limitations including tumor heterogeneity and difficulty in obtaining samples. As a non-invasive liquid biopsy capable of identifying molecular changes, genomic analysis of circulating tumor DNA released from cancer cells into blood has been proposed to determine real-time genomic landscapes of the tumor without additional burden and risk to patients [23].

Although several studies have suggested that cfDNA from patients with cancer can be used to detect mutations with high sensitivity [24-26], the detection of samples with low mutation fractions has uncertain clinical significance [27]. The study by Zhang et al. [20] demonstrated the feasibility of applying the ddPCR system to detect EGFR mutation and the advantage of ddPCR in the detection of samples with low EGFR mutation fractions.

In the present study, we demonstrated 74.1% sensitivity and 100% specificity of a plasma assay for EGFR mutations using ddPCR. The concordance rates of L858R and ex19del were 87.9% and 86.2%, respectively. Our results are consistent with those from previous studies [28, 29] and support the use of this technology in clinical practice for making treatment decisions especially for patients not amenable to tissue biopsy. The relatively lower clinical sensitivity of EGFR mutations in patients at the time of disease progression (65%) is likely attributed to the tumor shrinkage after drug treatment and the collection time of plasma samples near or at the PD stage.

In the current study with a total of 40 patients whose plasma collections from baseline were available for a longitudinal analysis, we observed a marked decrease in plasma EGFR mutations during the first 2 months in all patients after EGFR TKI treatment. More intriguingly, 34 patients (85%) had EGFR level decreased to undetectable level with longer PFS and OS than those with detectable EGFR in blood. In addition, the levels of both EGFR primary and resistance mutations in plasma increased progressively during disease progression. These findings suggest that longitudinal monitoring of plasma EGFR mutation levels can be a good predictive biomarker. Sorensen et al. [30] and Mok et al. [31] also showed the predictive value of serial assessment of cfDNA during treatment. To the best of our knowledge, this is the first study that evaluates longitudinal measurements of tumor volume with serial assessments of tumor DNA in blood for NSCLC patients treated with EGFR TKIs. The trend of mutation titer in plasma was well correlated with the tumor volume as measured by CT scan.

EGFR T790M is one of the most common mutations associated with acquired resistance to EGFR TKIs in NSCLC patients, which accounts for 50-60% [11, 32]. At present, the presence of T790M mutation is determined upon taking a new biopsy of the tumor from the patients at the time of progression. Given the limitations of repeated invasive biopsies, several studies were investigated to analyze acquired resistance to cancer therapies by non-invasive methods using plasma DNA [29, 33, 34]. We found that 14 patients (28.6%) among a total of 49 patients harbored resistance mutation T790M in the blood during EGFR TKIs treatment, which is similar to the study by Sakai et al. [35] in which T790M mutation was detected in 21 (30%) of 75 plasma samples from patients with clinical PD using the single allele base extension reaction (SABER) assay. It is noteworthy that the T790M mutant alleles were detectable in the blood of EGFR TKIs-treated patients as early as 12 months before radiographic documentation of disease progression in our study. This suggests that cfDNA may have important clinical implications as a useful surrogate biomarker of therapeutic response and be used to identify the development of resistant mutation, especially in early detection before it becomes clinically detectable. Furthermore, the detection of T790M mutation in cfDNA would be clinically useful for patients who are not amendable to repeated biopsies. Given the promising clinical results of several 3^rd^ generation EGFR TKIs in patients with T790M mutation [36-38], these treatment therapies will soon be available and accessible warranting effective clinical diagnostics to evaluate drug resistance. However, it still remains undetermined whether T790M mutation detected in cfDNA prior to clinically overt progression will change treatment decisions because patients harboring this mutation are usually indolent and EGFR TKI can be continued beyond progression [39]. Therefore, the clinical impact of early detection of the resistance mutation in plasma DNA needs to be elucidated in the near future.

In conclusion, this study demonstrated that plasma EGFR assay using ddPCR can be feasible and effective in monitoring treatment response to EGFR TKIs, and detecting the underlying mechanism of resistance in a noninvasive method. Determining the genetic landscapes of tumors through noninvasive real-time mutational profiling may have significant clinical implications in identifying and guiding optimal treatment strategies, particularly when T790M mutation is detected in the blood.

## MATERIALS AND METHODS

### Study design

This was an exploratory study to evaluate the potential utilization of EGFR mutations in plasma using ddPCR in monitoring treatment response and resistance among NSCLC patients harboring EGFR activating mutations treated with EGFR TKIs. Eligible patients had a diagnosis of advanced or recurrent NSCLC harboring activating EGFR mutation (exon 19 deletion or L858R) who had progressed on gefitinib or erlotinib. The analysis of EGFR (exon 18-21) mutations for tumor tissues obtained from either diagnostic or surgical procedures was performed prior to TKI treatment by direct sequencing or PNA-clamp method (Panagene Inc., Daejeon, Korea). Patients were treated with gefitinib (250 mg po daily) or erlotinib (150 mg po daily) at the discretion of treating physicians. Appropriate imaging measures including CT scans of the chest were performed every two cycles (8 weeks) and evaluated treatment response according to Response Evaluation Criteria in Solid Tumors (RECIST version 1.1). All patients provided written informed consent. This study was performed in accordance with the Declaration of Helsinki and approved by Institutional Review Board for Human Research at each institute.

The primary objective of this exploratory analysis was to assess the diagnostic utility of the blood test for sensitivity, specificity, concordance rate, and comparison with tumor tissue EGFR mutation status. The secondary objectives included identifying the time difference between development of genetic aberrancy associated with resistance in plasma and overt clinical progression.

### Sample collection and DNA extraction

Plasma samples were prospectively obtained before treatment and every 8 weeks post-treatment until development of disease progression for cell-free DNA (cfDNA) EGFR ddPCR testing. Blood was collected into one 10-mL EDTA-containing vacuum tube and was centrifuged at 1500 rpm for 15 minutes within 4 hours of collection. The supernatants were collected and centrifuged at 1500 rpm for 15 minutes. The plasmas were transferred to 1.5-mL Eppendorf tubes and stored at −70°C until DNA extraction. The cfDNA was extracted from 1-2 mL of plasma using QIAamp Circulating Nucleic Acid Kit (Qiagen) according to the manufacturer's instructions.

### Droplet digital PCR detection of EGFR mutations in plasma cfDNA

Primers and probes were custom synthesized by Thermo Fisher and the sequences were reported previously [28]. Briefly, the ddPCR assay was conducted by droplets generation using QX200 generator (Bio-Rad Laboratories, Inc., Hercules, CA, USA), followed by endpoint PCR reactions using C1000 (Bio-Rad) and droplet flow cytometry readings using QX200 reader (Bio-Rad). Analyzed data were processed using QuantaSoft (Bio-Rad) software. Ex19del assay covers all deletion mutations in the exon 19. Detailed procedures can be found elsewhere [20].

### Measurement of tumor volume

Considering the recent studies that the conventional RECIST-based assessment alone does not fully characterize response and progression in genomically characterized patients [40, 41], we further explored the volumetric tumor response during TKI therapy. All CT image data were reconstructed with a section thickness of 1.25 mm using soft-tissue algorithms. For each measurable lesion, volume measurements were performed with a semi-automated segmentation method using MRIcro (version 1.40, Chris Rorden, University of Nottingham, Great Britain). Using in-house software, the computer automatically calculated the volume (cm^3^) by multiplying the number of voxels segmented by the unit volume of a voxel. A sum of the volume for all target lesions at each time-point was used for the analysis.

### Statistical analysis

Sensitivity was calculated as the number of samples positive in both tissue and plasma out of the positive tissue samples, whereas specificity was calculated from the number of plasma- and tissue-negative samples out of the total negative tissue samples. Concordance rate was calculated as the number of samples positive in both tissue and plasma, plus the number of samples negative in both tissue and plasma, out of the total number of matched samples. Survival estimates were calculated according to Kaplan-Meier method. Cox regression methods were used for biomarker at baseline associated with PFS. Statistical analysis was performed using SAS 9.4 (SAS Institute Inc, Cary, NC) and R version 3.0.3 (Vienna, Austria; http://www.R-project.org/). A 2-sided *P*-value of < 0.05 was considered statistically significant.

## SUPPLEMENTARY MATERIAL FIGURES


