# Supplementary material for: Longitudinal monitoring of EGFR mutations in plasma predicts outcomes of NSCLC patients treated with EGFR TKIs: Korean Lung Cancer Consortium (KLCC-12-02)
Source: Oncotarget. 2016 Jan 9;7(6):6984–93. doi: 10.18632/oncotarget.6874 (PMC4872763; doi:10.18632/oncotarget.6874)
Supplement: Supplementary file 1 [file oncotarget-07-6984-s001.pdf]

Longitudinal monitoring of EGFR mutations in plasma predicts outcomes of NSCLC patients treated with EGFR TKIs: Korean Lung Cancer Consortium (KLCC-12-02)

Supplementary Material

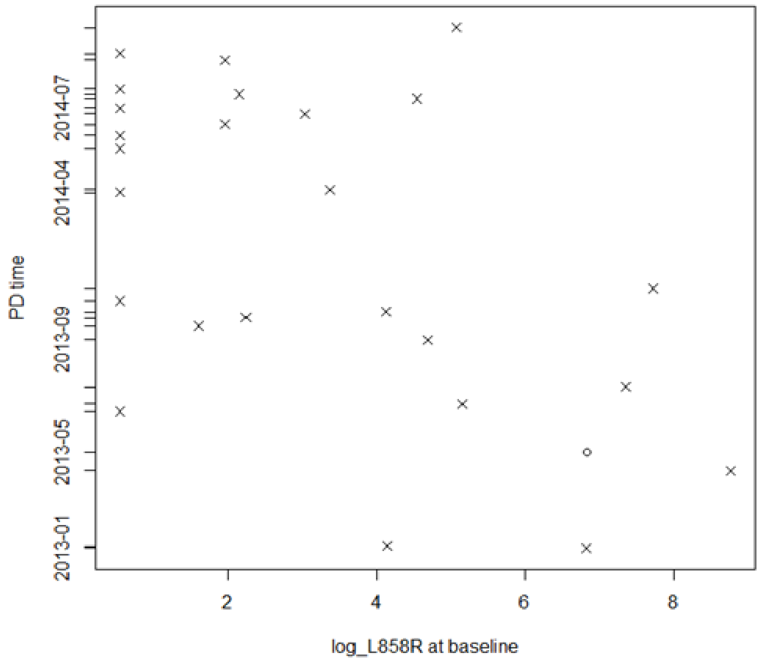

	coef	exp(coef)	se(coef)	z	Pr(>|z|)
log_L858R at baseline	0.25724	1.29336	0.09498	2.708	0.00676

Supplementary Figure 1. Correlation between the level of L858R copies at baseline and time of disease progression.

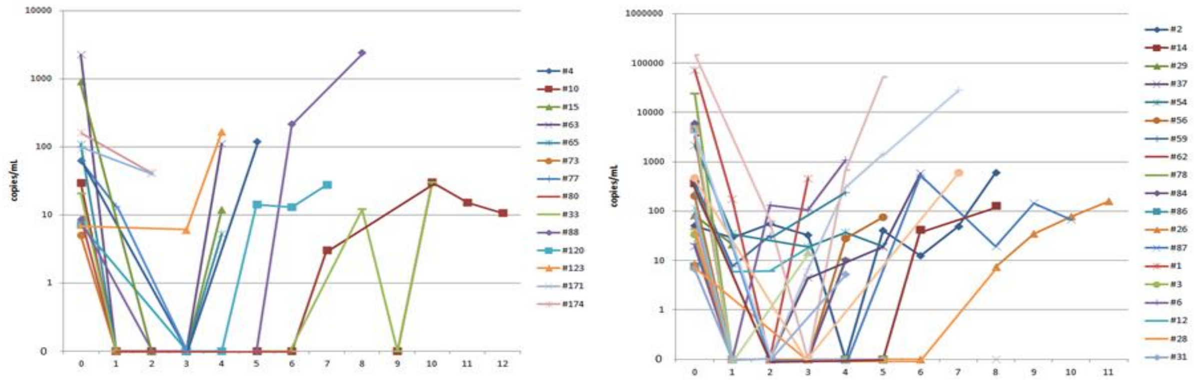

Supplementary Figure 2. Copy number changes for 40 patients L858R (A) and ex19del (B)

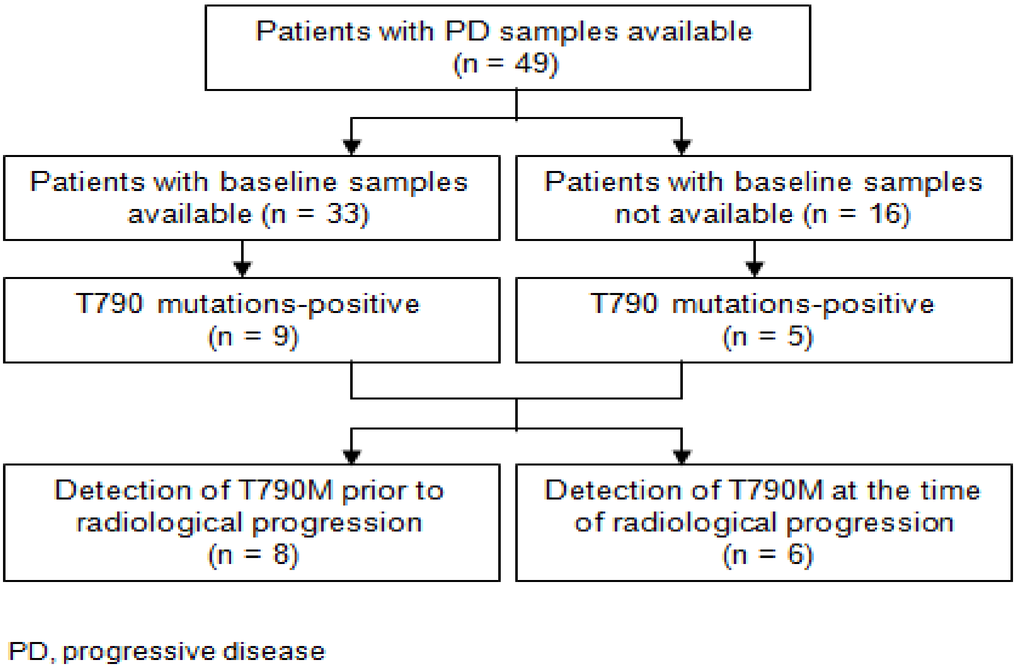

Supplementary Figure 3. Patient flow chart for T790M mutation
